# Antimycobacterial and PknB Inhibitory Activities of Venezuelan Medicinal Plants

**DOI:** 10.1155/2020/8823764

**Published:** 2020-08-01

**Authors:** C. A. Aranaga, S. Fraile, A. Torres, A. Falco, F. Michelangeli, H. Takiff

**Affiliations:** ^1^Grupo de Investigación en Química y Biotecnología, Facultad de Ciencias Básicas, Universidad Santiago de Cali, Cali, Colombia; ^2^Laboratorio de Genética Molecular, Instituto Venezolano de Investigaciones Científicas, Caracas, Venezuela; ^3^Centro de Biofísica y Bioquímica, Instituto Venezolano de Investigaciones Científicas, Caracas, Venezuela; ^4^Grupo de Investigación en Microbiología Industria y Ambiente, Universidad Santiago de Cali, Cali, Colombia; ^5^Integrated Mycobacterial Pathogenomics, Institut Pasteur, Paris, France

## Abstract

Global control and elimination of tuberculosis are hindered by the high prevalence of drug-resistant strains, making the development of new drugs to fight tuberculosis a public health priority. In this study, we evaluated 118 extracts from 58 Venezuelan plant species for their ability to inhibit the growth of *Mycobacterium tuberculosis* mc^2^6020, using the agar dilution method. Additionally, we determined the ability of these extracts to inhibit the activity of PknB protein, an essential *M. tuberculosis* serine/threonine kinase, using a high-throughput luminescent assay. Of the 118 extracts tested, 14 inhibited bacterial growth with a minimum inhibitory concentration ≤500 *μ*g/ml, and 36 inhibited the kinase activity with a half-maximal inhibitory concentration <200 *μ*g/ml. Five extracts inhibited *M. tuberculosis* growth and inhibited the activity of the kinase protein, suggesting that this could be the basis of their growth inhibition.

## 1. Introduction

According to the data from the World Health Organization (WHO), 10 million people developed tuberculosis (TB) in 2018, while 1.5 million died from this disease [1]. In 2014, the WHO launched the “END TB,” strategy, which seeks to end the global tuberculosis epidemic. One of the pillars of this strategy is the search for new treatment alternatives for both drug-sensitive and multidrug-resistant tuberculosis strains.

Just three new compounds, bedaquiline, delamanid, and pretomanid, have received approval for use against TB [2, 3]. Although they appear to have good anti-TB activity and could be useful for treating strains resistant to other agents, their potential for side effects and the possible emergence of resistant strains [4, 5] have led to caution and reservations about their appropriate role in treatment regimens. Therefore, the search for new drugs that are safe and effective in the control of TB continues to be a priority.

Recent studies have shown the potential of natural extracts as the basis for new drugs [6, 7]. Microorganisms and plants have exceptional chemical and structural diversity and represent a valuable source of biologically active molecules with potential as new antimicrobials [8]. For example, ursolic acid and hydroquinone, isolated from methanolic extracts of *Artemisia capillaris*, inhibit the growth of susceptible and resistant strains of *M. tuberculosis* and have promise as potential chemotherapeutic agents [9]. Ethnopharmacological research helps to focus screening efforts by identifying groups of plants that are used in traditional medicine to treat infectious diseases. Screening performed with extracts of medicinal plants used in India, Mexico, and South Africa [10–12] demonstrated that some of these extracts have high activity for inhibiting the growth of *M. tuberculosis* and other mycobacteria.

In Venezuela, many indigenous and local communities continue to use plants for medicinal purposes, but few of these plants have been evaluated for their antituberculosis activity. We were particularly interested in extracts that inhibited the activity of PknB, a serine/threonine kinase that appears to be essential in *Mycobacteria*. However, compounds found to inhibit enzyme activity with *in vitro* screens can, for a variety of reasons, fail to inhibit the growth of the whole bacteria. Therefore, in this study, we selected 118 extracts from 58 plant species used in traditional Venezuelan medicine and tested their ability to inhibit the *in vitro* activity of the *M. tuberculosis* serine/threonine-protein kinase PknB and also their ability to inhibit the growth of *M. tuberculosis* mc^2^6020.

## 2. Materials and Methods

### 2.1. Plant Material

The extracts tested were obtained from plants in the botanical species collection (MedPlant) [13] of the Gastrointestinal Physiology Laboratory of Instituto Venezolano de Investigaciones Científicas (IVIC), which is covered by an “Access Contract to Genetic Resources” signed between IVIC and the Ministry of Environment and Natural Resources.

**Figure 1:**
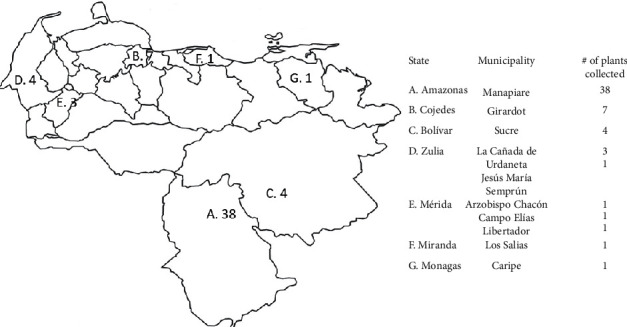
Plant collection sites. The plants were collected in 7 states of Venezuela, the main collection site being the Amazon state.

A total of 118 ethanolic extracts were obtained from 58 plant species, belonging to 45 genera and 33 families (see Table S1 in the Supplementary Material). The plants were collected in the Venezuelan states of Amazonas, Bolívar, Cojedes, Mérida, Miranda, and Monagas, as shown in Figure 1. The extracts include different plant organs, such as bark, branch, flower, fruit, and leaf. Plants were selected according to their ethnopharmacological activity for the treatment of symptoms associated with lung diseases (cough, fever, nasal congestion, and asthma) from the PlantMed database, which contains data on Venezuelan and neotropical plants reported as having medicinal use.

The plant materials were dried at room temperature. After grinding in a blender, plant material was macerated in three volumes of 95% ethanol for at least 3 days. The material was filtered, and the alcoholic extract was concentrated and dried by a rotary evaporator and lyophilization. Stock extract solutions (100 mg/ml) were prepared in 100% dimethyl sulfoxide (DMSO) immediately before use.

### 2.2. Strain and Growth Conditions

*M. tuberculosis* mc^2^6020 was grown in 7H9 and 7H11 Middlebrook media to which was added 10% ADC (0.5% bovine serum albumin, 0.2% glucose, and 0.85% NaCl), L-lysine (40 *μ*g/ml), and pantothenic acid (20 *μ*g/ml). The 7H9 medium additionally contained Tween-80 (0.5% v/v) and glycerol (0.2% v/v) [14]. The strain was incubated at 37°C in an incubator-shaker until reaching an optical density at 600 nm (OD_600mn_) of 0.6 (approximately 3 × 10^8^ CFU/ml) and then diluted in a 1 : 2,000 ratio for studies of growth inhibition. Cultures of *Escherichia coli* BL21 (DE3 pLys) were cultured at 37°C in LB broth in an incubator-shaker until reaching an OD_600nm_ of 0.6.

### 2.3. Antituberculosis Screening

The antituberculosis activity of the extracts was determined using the agar dilution method. For this, 35 × 10 mm Petri dishes were prepared with medium supplemented with a plant extract at a final concentration of 500 *μ*g/ml. Petri dishes with 0.5% DMSO but without extracts were prepared as growth controls, while as a positive control, plates supplemented with Kanamycin (25 mg/ml) were used. *M. tuberculosis* mc^2^6020 cultures (10 *μ*l) were spread onto the medium, and then, the Petri dishes were incubated at 37°C for 25 to 30 days, or until growth was observed in the control without extract. The extract containing plates on which no growth was seen was taken to indicate growth inhibition, and the minimum inhibitory concentration (MIC) was established by repeating the above procedure with medium containing 100, 200, 300, 400, and 500 *μ*g/ml.

### 2.4. Recombinant Protein Expression and Purification

The 279-residue kinase domain of PknB (DQPknB) [15] and full-length GarA [16], natural substrates for the PknB protein, were expressed in *Escherichia coli* BL21 (DE3 pLys). Cultures were grown until an OD_600nm_ of 0.6 and induced by the addition of IPTG (final conc. 1 mM), incubated at 37°C for an additional eight hours and then centrifuged at 1,503 g. The pellet was resuspended in lysis buffer (50 mM of Tris-HCl, 500 mM of NaCl, and 20 mM of imidazole, at a pH of 7.5) in the presence of lysozyme and DNase I from bovine pancreas (Sigma, St Louis, MO, USA). The bacteria were sonicated, and the resulting lysate was centrifuged at 18,407 g for 15 min. The supernatant was passed through a 0.45 *μ*m syringe filter and then loaded onto a Ni Sepharose™ high-performance column (Amersham) using a fast protein liquid chromatography (FPLC) system. The elution was performed with an imidazole gradient (0.0–0.5 M), and the fractions containing the protein of interest were visualized by sodium dodecyl sulfate-polyacrylamide gel electrophoresis (SDS-PAGE).

### 2.5. Phosphorylation Test

Phosphorylation assays were carried out using the commercial Kinase Glo kit (Promega) in a final volume of 100 *μ*l in 96-well solid white polystyrene microplates. The concentrations of ATP (10 *μ*M), DQPknB (0.056 *μ*M), and GarA (0.48 *μ*M) were standardized using the manufacturer's recommendations. All reactions were carried out at room temperature and were started with reaction buffer mixture (200 mM of Tris, 8 mM of MnCl_2_, 10 mM of KF, and 1 mM of DTT), GarA, DQPknB, and the extract (10, 30, 65, 125, 250, and 500 *μ*g/ml). A negative control was elaborated by replacing the volume of the extract with DMSO. After 10 min, ATP was added, and the reaction was incubated for an additional 10 min. At the end of this period, the reaction was stopped by the addition of 50 *μ*l of the Kinase Glo reagent, and after another 10 min, the luminescent signal was measured on a SpectraMax Gemini XS microplate spectrofluorometer (Molecular Devices). Half-maximal inhibitory concentration (IC_50_) values were calculated using the GraphPad Prism 5 software.

## 3. Results

We evaluated the inhibitory activity of 118 extracts of Venezuelan medicinal plants on the *in vitro* activity of the PknB protein and on the growth of the *M. tuberculosis* mc^2^6020. The results obtained show that 14 extracts (11.76%) inhibited the growth of the bacteria at a concentration ≤500 *μ*g/ml (Table 1).

The leaf extract of the species *Piper sanvicentense* exhibited the best activity against the growth of *M. tuberculosis* mc^2^6020 with an MIC of 100 *μ*g/ml, followed by the inner bark of *Eschweilera parvifolia* with an MIC of 300 *μ*g/ml and extracts of inner bark and branch of *Protium heptaphyllum* with an MIC of 400 *μ*g/ml. All other growth inhibiting extracts (*Piper marginatum* (stem), *Macoubea guianensis* (leaf), *Senna silvestris* (flower), *Bromelia goeldiana* (fruit), *Polypodium aureum* (stem), *Byrsonima crassifolia* (flower), *Hyptis dilatata* (flower and root), *Xylopia aromatica* (leaf), and *Curatella americana* (leaf)) showed MICs of 500 *μ*g/ml.

Of the 36 extracts capable of inhibiting the *in vitro* activity of DQPknB, four had an IC_50_ lower than 50 *μ*g/ml, while 19 had an IC_50_ between 50 and 100 *μ*g/ml and the remaining 13 exhibited an IC_50_ between 100 and 200 *μ*g/ml (Table 2).

Six extracts (5.04%) were found to inhibit both the *in vitro* activity of PknB and the growth of *M. tuberculosis* mc^2^6020 (3), the inner bark of *Eschweilera parvifolia* and *Protium heptaphyllum*, the branch of *Protium heptaphyllum*, the flower of *Byrsonima crassifolia,* and the young leaves of *Curatella americana* and *Xylopia aromatica*.

## 4. Discussion

In this study, we selected 58 species of Venezuelan medicinal plants used by indigenous communities for the treatment of respiratory symptoms and evaluated extracts of different plant parts in a screen for natural compounds that have potential as antituberculosis. We tested a total of 118 extracts of different plant parts and of the 14 that inhibited the growth of *M. tuberculosis* mc^2^6020 and 36 that inhibited the *in vitro* activity of PSTK PknB, and there were five that had both inhibitory activities.

The plants that showed activity against growth were *P. sanvicentense* (Piperaceae), *E. parvifolia* (Lecythidaceae)*, Pro. heptaphyllum* (Burseraceae)*, Brom. goeldiana* (Bromeliaceae), *B. crassifolia* (Malpighiaceae), *C. americana* (Dilleniaceae), *H. dilatata* (Lamiaceae), *M. guianensis* (Apocynaceae), *P. marginatum* (Piperaceae), *Pol. aureum* (Polypodiaceae), *S. silvestris* (Caesalpiniaceae), and *X. aromatica* (Annonaceae).

Our finding that an ethanolic extract bark of *C. americana* inhibited *M. tuberculosis* growth with an MIC of 500 *μ*g/ml agreed with the results of Pavan et al. [17], who found that a methanolic extract of the bark inhibited the growth of *M. tuberculosis* H37Rv with the same MIC (Table 1), thus confirming the presence of compounds with antituberculosis activity in this species. We also tested extracts of young leaves, leaves, flowers, and branches of *C. Americana,* but these had MICs that were higher than 500 *μ*g/ml (data not shown), suggesting a differential distribution of the active metabolites throughout the plant. All *C. americana* extracts also showed activity against DQPknB, with an IC_50_ lower than 200 *μ*g/ml. However, while the lowest MIC for growth inhibition was in the bark extract, the extract with the lowest IC_50_ for inhibiting PknB activity was from young leaves, with an IC_50_ of 39.5 *μ*g/ml, while that of the bark was 124.9 *μ*g/ml.

Similarly, the plant parts of *H. dilatata* and *E. parvifolia* whose extracts had the greatest capacity to inhibit kinase activity were not the parts of these plants that were best at inhibiting bacterial growth (Tables 1 and 2). This could suggest that the molecules that inhibit kinase activity are different from those that mediate growth inhibition. Another possibility is that molecules that inhibit kinase activity *in vitro* have trouble penetrating the bacterial cell wall to inhibit the enzyme in live bacteria. Nevertheless, our study shows that *C. americana, H. dilatata,* and *E*. *parvifolia* are promising species for molecules with antituberculosis potential.

The stem extract from *P. marginatum* inhibited *M. tuberculosis* growth with an MIC of 500 *μ*g/ml. Ethanolic extracts of *P. marginatum* have been previously shown to inhibit the growth of *Porphyromonas gingivalis* ATCC 33277, *Fusobacterium nucleatum* ATCC 25586, and *Prevotella intermedia* ATCC 25611 [18]. *P. marginatum* essential leaf oil showed a bactericidal effect against *Xanthomonas albilineans* and *X. campestris* pv. *campestris* and a fungistatic effect against *Alternaria solani* [19]. Terpenoid compounds have shown to have moderate to high activity against *M. tuberculosis*, and the presence of these compounds in *P. marginatum* could explain their antituberculosis and antimicrobial activity [18].

Previous research established the potential of the ethanolic extract of *P. sanvicentense* against breast cancer MDA-MB-231, prostate cancer PC-3, and human colon cancer HT29 cell lines [13], but this is the first report of its antibacterial activity. Of the 118 extracts we tested, the *P. sanvicentense* extract had lowest MIC for inhibiting *M. tuberculosis* growth (100 *μ*g/ml). Although it did not show any ability to inhibit kinase activity, *P. sanvicentense* could be a promising candidate for chemical analysis. This is also the first report of antibacterial activity in the species *Brom. goeldiana*, *M. guianensis*, and *Pol. aureum*, whose fruit, leaf, and stem extracts, respectively, showed activity only against bacterial growth at an MIC of 500 *μ*g/ml.

Although antibacterial activity has previously been described in *B. crassifolia*, *Pro. heptaphyllum,* and *Xylopia aromatica* [20–24], we found that only some of the plant part extracts inhibited both *M. tuberculosis* growth and the kinase activity. The flower extract of *B. crassifolia* had an IC_50_ of 30.4 *μ*g/ml and an MIC of 500 *μ*g/ml, while the inner bark extract showed an IC_50_ of 144 *μ*g/ml but an MIC higher than 500 *μ*g/ml. Similarly, an extract of the inner bark of *Pro. heptaphyllum* had an IC_50_ of 37.4 *μ*g/ml and an MIC of 400 *μ*g/ml, but the leaf extract had an IC_50_ of 95.7 *μ*g/ml and an MIC over 500 *μ*g/ml. The young leaf extract of *Xylopia aromatica* inhibited both growth and kinase activity (MIC = 500 *μ*g/ml and IC_50_ = 93.6 *μ*g/ml), while the other extracts of this plant showed neither activity. Terpenoid compounds have been shown to possess high antituberculosis activity [25]. Phytochemical studies of the essential oils of *X. aromatica* and *Prot. heptaphyllum* leaves demonstrated the presence of various terpenes, including spathulenol [20, 21]. This sesquiterpene has been shown to have antibacterial activity against *Cryptococcus neoformans*, *Enterococcus faecalis*, and *Staphylococcus aureus* [26].

The extracts of *Jacaranda copaia*, *Oenocarpus bataua*, *Parkia pendula*, *Protium crassipetalum*, *Tapirira guianensis*, *Uncaria guianensis, Vismia cayennensis, Vismia guianensis, Vochysia ferrugínea, Waltheria indica*, and *Warscewiczia coccinea* inhibited the kinase, but not mycobacterial growth. Nevertheless, identification of the kinase inhibiting metabolites could provide new lead compounds for the design of new drugs.

The search for new antituberculosis drugs continues to be a challenge, but the high incidence of this disease makes it an important priority. In this study, we documented the antimycobacterial potential of some Venezuelan plants for which antibacterial activity has not been previously reported and demonstrated the importance of ethnopharmacological knowledge in the search for botanical sources with biological activity. To our knowledge, this is the first study demonstrating the *in vitro* activity of natural extracts on the activity of the catalytic domain of the *M. tuberculosis* protein PknB. Serine/threonine kinases are important targets for anticancer agents [27] and have been proposed as targets for antituberculosis drugs. PknB is essential for *M. tuberculosis* [28], but no drug targeting it has yet been identified. In the extracts, we identified that plant extracts inhibit both *M. tuberculosis* growth and the kinase, and it is tempting to believe the same molecules contain both inhibitory activities, but this must be proven when the active compounds are successfully isolated and identified.

## 5. Conclusions

*P. sanvicentense* (Piperaceae), *E. parvifolia* (Lecythidaceae), *Pro. heptaphyllum* (Burseraceae), *Brom. goeldiana* (Bromeliaceae), *B. crassifolia* (Malpighiaceae), *C. americana* (Dilleniaceae), *H. dilatata* (Lamiaceae), *M. guianensis* (Apocynaceae), *P. marginatum* (Piperaceae), *Pol. aureum* (Polypodiaceae), *S. silvestris* (Caesalpiniaceae), and *X. aromatica* (Annonaceae) total ethanol extracts presented outstanding antimicrobial activity against *M. tuberculosis* mc^2^6020.

Of the 36 extracts that were shown to inhibit the activity of the PknB protein, only 5 were able to inhibit the growth of *M. tuberculosis*. Further studies should be performed to determine whether compounds that inhibit the activity of protein kinase also affect bacterial growth.

## Figures and Tables

**Table 1:** Antimycobacterial activity of plant extracts against *M. tuberculosis* mc^2^6020.

Botanical species (family)	Voucher/collection number	Part	MIC (*μ*g/ml)
*Piper sanvicentense* (Piperaceae)	25263 AF	Leaf	100
*Eschweilera parvifolia* (Lecythidaceae)	01441 BM	Inner bark	300
*Protium heptaphyllum* (Burseraceae)	16278 AF	Inner bark	400
*Protium heptaphyllum* (Burseraceae)	16278 AF	Branch	400
*Bromelia goeldiana* (Bromeliaceae)	01488 BM	Fruit	500
*Byrsonima crassifolia* (Malpighiaceae)	00485 BM	Flower	500
*Curatella americana* (Dilleniaceae)	01891 BM	Bark	500
*Hyptis dilatata* (Lamiaceae)	01036 BM	Flower	500
*Hyptis dilatata* (Lamiaceae)	00507 BM	Root	500
*Macoubea guianensis* (Apocynaceae)	00582 BM	Leaf	500
*Piper marginatum* (Piperaceae)	00485 BM	Stem	500
*Polypodium aureum* (Polypodiaceae)	00834 BM	Stem	500
*Senna silvestris* (Caesalpiniaceae)	00507 BM	Flower	500
*Xylopia aromatica* (Annonaceae)	14612 AF	Young leaf	500

**Table 2:** The activity of plant extracts against DQPknB of *M. tuberculosis*.

Botanical species (family)	Voucher/collection number	Part	IC_50_ (*μ*g/ml)
*Byrsonima crassifolia* (Malpighiaceae)	00485 BM	Flower	30.4
*Protium heptaphyllum* (Burseraceae)	16278 AF	Internal bark (root)	37.4
*Senna silvestris* (Caesalpiniaceae)	00507 BM	Leaf	37.4
*Curatella americana* (Dilleniaceae)	01053 BM	Young leaf	39.5
*Cochlospermum orinocense* (Bixaceae)	01473 BM	Stem	55
*Eschweilera parvifolia* (Lecythidaceae)	01441 BM	Branch	58.8
*Vismia guianensis* (Clusiaceae)	01523 BM	Leaf	58.8
*Waltheria indica* (Sterculiaceae)	16607 AF	Whole plant without root	59.1
*Vochysia ferruginea* (Vochysiaceae)	01286 BM	Stem	59.5
*Oenocarpus bataua* (Arecaceae)	01524 BM	Root	60.9
*Humiria balsamífera* (Humiriaceae)	00189 BM	Leaf	64.7
*Protium heptaphyllum* (Burseraceae)	16278 AF	Branch	66
*Hyptis dilatata* (Lamiaceae)	00507 BM	Root	66.6
*Uncaria guianensis* (Rubiaceae)	01525 BM	Bark	71.2
*Vismia guianensis* (Clusiaceae)	01523 BM	Bark	75.2
*Eschweilera parvifolia* (Lecythidaceae)	01441 BM	Internal bark (root)	76.5
*Euterpe precatoria* (Arecaceae)	01690 BM	Root	77.4
*Warscewiczia coccinea* (Rubiaceae)	01316 BM	Stem	79
*Protium crassipetalum* (Burseraceae)	01409 BM	Bark	79.7
*Curatella americana* (Dilleniaceae)	01053 BM	Flower	81.9
*Hyptis dilatata* (Lamiaceae)	00507 BM	Leaf, branch without flower	81.9
*Xylopia aromatica* (Annonaceae)	14612 AF	Young leaf	93.6
*Protium heptaphyllum* (Burseraceae)	16278 AF	Leaf	95.7
*Croton cuneatus* (Euphorbiaceae)	01247 BM	Young leaf	124.9
*Curatella americana* (Dilleniaceae)	01053 BM	Bark	124.9
*Vochysia ferrugínea* (Vochysiaceae)	01286 BM	Leaf	130.9
*Byrsonima crassifolia* (Malpighiaceae)	00485 BM	Internal bark (root)	144
*Cochlospermum orinocense* (Bixaceae)	01473 BM	Flower	144.2
*Curatella americana* (Dilleniaceae)	01053 BM	Leaf	146
*Cochlospermum orinocense* (Bixaceae)	01473 BM	Leaf	156.9
*Parkia pendula* (Mimosaceae)	00414 BM	Bark	158.3
*Croton cuneatus* (Euphorbiaceae)	01250 BM	Internal bark (root)	161.7
*Tapirira guianensis* (Anacardiaceae)	14733 AF	Flower	164.4
*Jacaranda copaia* (Bignoniaceae)	22150 AF	Young leaf	174.1
*Eschweilera parvifolia* (Lecythidaceae)	01441 BM	Leaf	183.3
*Vismia cayennensis* (Clusiaceae)	01484 BM	Leaf	185.8

**Table 3:** Extracts that showed activity against DQPknB and *M. tuberculosis* mc^2^6020.

Botanical species	Part	IC_50_	CMI
*Eschweilera parvifolia*	Internal bark (root)	76.53	300
*Protium heptaphyllum*	Internal bark (root)	37.40	400
*Protium heptaphyllum*	Branch	66.05	400
*Byrsonima crassifolia*	Flower	30.41	500
*Curatella americana*	Young leaf	146.04	500
*Xylopia aromatica*	Young leaf	93.63	500

## Data Availability

The data used to support the findings of this study are included within the article and are available from the corresponding author upon request.

## Supplementary Materials

Supplementary Materials Table S1: medicinal plant extracts evaluated in this study.

## References

[1] World Health Organization (2019). *Global TB Report 2019*.

[2] Brigden G., Hewison C., Varaine F. (2015). New developments in the treatment of drug-resistant tuberculosis: clinical utility of bedaquiline and delamanid. *Infection and Drug Resistance*.

[3] Keam S. J. (2019). Pretomanid: first approval. *Drugs*.

[4] Fujiwara M., Kawasaki M., Hariguchi N., Liu Y., Matsumoto M. (2018). Mechanisms of resistance to delamanid, a drug for *Mycobacterium tuberculosis*. *Tuberculosis*.

[5] Nguyen T. V. A., Anthony R. M., Bañuls A.-L., Nguyen T. V. A., Vu D. H., Alffenaar J.-W. C. (2017). Bedaquiline resistance: its emergence, mechanism, and prevention. *Clinical Infectious Diseases*.

[6] Harvey A. L., Edrada-ebel R., Quinn R. J. (2015). The re-emergence of natural products for drug discovery in the genomics era. *Nature Reviews Drug Discovery*.

[7] Quan D., Nagalingam G., Payne R., Triccas J. A. (2017). New tuberculosis drug leads from naturally occurring compounds. *International Journal of Infectious Diseases*.

[8] Payne D. J., Gwynn M. N., Holmes D. J., Pompliano D. L. (2007). Drugs for bad bugs: confronting the challenges of antibacterial discovery. *Nature Reviews Drug Discovery*.

[9] Jyoti M. A., Nam K.-W., Jang W. S. (2016). activity of methanolic plant extract of Artemisia capillaris containing ursolic acid and hydroquinone against *Mycobacterium tuberculosis*. *Journal of Infection and Chemotherapy*.

[10] Gupta V. K., Kaushik A., Chauhan D. S., Ahirwar R. K., Sharma S., Bisht D. (2018). Anti-mycobacterial activity of some medicinal plants used traditionally by tribes from Madhya Pradesh, India for treating tuberculosis related symptoms. *Journal of Ethnopharmacology*.

[11] Mativandlela S. P. N., Meyer J. J. M., Hussein A. A., Houghton P. J., Hamilton C. J., Lall N. (2008). Activity againstMycobacterium smegmatis and *M. tuberculosis* by extract of South African medicinal plants. *Phytotherapy Research*.

[12] Jimenez-Arellanes A., Meckes M., Ramirez R., Torres J., Luna-Herrera J. (2003). Activity against multidrug-resistant *Mycobacterium tuberculosis* in Mexican plants used to treat respiratory diseases. *Phytotherapy Research*.

[13] Taylor P., Arsenak M., Abad M. J. (2013). Screening of Venezuelan medicinal plant extracts for cytostatic and cytotoxic activity against tumor cell lines. *Phytotherapy Research*.

[14] Bardarov S., Pavelka M. S., Sambandamurthy V. (2002). Specialized transduction: an efficient method for generating marked and unmarked targeted gene disruptions in *Mycobacterium tuberculosis*, *M. bovis* BCG and *M. smegmatis*. *Microbiology*.

[15] Boitel B., Ortiz-Lombardía M., Durán R. (2003). PknB kinase activity is regulated by phosphorylation in two Thr residues and dephosphorylation by PstP, the cognate phospho-Ser/Thr phosphatase, in *Mycobacterium tuberculosis*. *Molecular Microbiology*.

[16] Villarino A., Duran A., Wehenkel P. (2005). Proteomic identification of M.tuberculosis protein kinase substrates: PknB recruits GarA, a FHA domain-containing protein, through activation loop-mediated interactions. *Journal of Molecular Biology*.

[17] Pavan F. R., Sato D. N., Higuchi C. T., Santos A. C. B., Vilegas W., Leite C. Q. E. (2009). In vitro anti-*Mycobacterium tuberculosis* activity of some Brazilian “Cerrado” plants. *Revista Brasileira de Farmacognosia*.

[18] Gamboa F., Muñoz C.-C., Numpaque G., Sequeda-Castañeda L. G., Gutierrez S. J., Tellez N. (2018). Antimicrobial activity ofPiper marginatumJacq andIlex guayusaLoes on microorganisms associated with periodontal disease. *International Journal of Microbiology*.

[19] Sánchez Y., Correa T. M., Abreu Y., Martínez B., Duarte Y., Pino O. (2011). Caracterización Química y Actividad Antimicrobiana del Aceite Esencial de Piper marginatum Jacq. *Rev. Protección Veg.*.

[20] Alcântara J. M., De Lucena J. M. V. M., Facanali R., Marques M. O. M., Da Paz Lima M. (2017). Chemical composition and bactericidal activity of the essential oils of four species of annonaceae growing in Brazilian Amazon. *Natural Product Communications*.

[21] Cabral R. S. C., Alves C. C. F., Batista H. R. F. (2018). Composition of essential oils from different parts of Protium heptaphyllum (Aubl.) Marchand and their in vitro antibacterial activity. *Natural Product Research*.

[22] Cáceres A., López B., Juárez X., del Aguila J., García S. (1993). Plants used in Guatemala for the treatment of dermatophytic infections. 2. evaluation of antifungal activity of seven American plants. *Journal of Ethnopharmacology*.

[23] Martínez-Vázquez M., González-Esquinca A. R., Cazares Luna L., Moreno Gutiérrez M. N., García-Argáez A. N. (1999). Antimicrobial activity of Byrsonima crassifolia (L.) H.B.K. *J. Ethnopharmacol.*.

[24] Mendes J. L., de Araújo T. F., Geraldo De Carvalho M., Aragão Catunda Júnior F. E., Albuquerque Costa R. (2019). Chemical composition and mechanism of vibriocidal action of essential oil from resin of Protium heptaphyllum. *The Scientific World Journal*.

[25] Ndjoubi K. O., Sharma R., Hussein A. A. (2020). The potential of natural diterpenes against tuberculosis: an updated review. *Current Pharmaceutical Design*.

[26] Fernandes F. H., Guterres Z. d. R., Violante I. M. P., Lopes T. F. S., Garcez W. S., Garcez F. R. (2015). Evaluation of mutagenic and antimicrobial properties of brown propolis essential oil from the Brazilian Cerrado biome. *Toxicology Reports*.

[27] Capdeville R., Buchdunger E., Zimmermann J., Matter A. (2002). Glivec (STI571, imatinib), a rationally developed, targeted anticancer drug. *Nature Reviews Drug Discovery*.

[28] Fernandez P., Saint-Joanis B., Barilone N. (2006). Ser/Thr protein kinase PknB is essential for sustaining mycobacterial growth. *Journal of Bacteriology*.

