# Supplementary material for: Antimycobacterial and PknB Inhibitory Activities of Venezuelan Medicinal Plants
Source: Int J Microbiol. 2020 Aug 1;2020:8823764. doi: 10.1155/2020/8823764 (PMC7416274; doi:10.1155/2020/8823764)
Supplement: Supplementary Materials — Table S1: medicinal plant extracts evaluated in this study. [file 8823764.f1.docx]

Table S1. Medicinal plant extracts evaluated in this study.

| **Family** | **Plant species** | **Part of plant** | **Collection site** | **Voucher #** |
| --- | --- | --- | --- | --- |
| *Anacardiaceae* | *Tapirira guianensis* | RB | Amaz/Man | 14733 AF |
| *Anacardiaceae* | *Tapirira guianensis* | FR | Amaz/Man | 14733 AF |
| *Annonaceae* | *Annona jahnii* | L | Coj/Gir | 01914 RG |
| *Annonaceae* | *Annona purpurea* | L | Bol/Suc | 01728 BM |
| *Annonaceae* | *Annona purpurea* | YL | Bol/Suc | 01728 BM |
| *Annonaceae* | *Annona purpurea* | RB | Bol/Suc | 01728 BM |
| *Annonaceae* | *Annona squamosa* | L | Bol/Suc | 01727 BM |
| *Annonaceae* | *Annona squamosa* | RB | Bol/Suc | 01727 BM |
| *Annonaceae* | *Guatteria schomburgkiana* | L | Amaz/Man | 00483 BM |
| *Annonaceae* | *Guatteria schomburgkiana* | FR | Amaz/Man | 00483 BM |
| *Annonaceae* | *Guatteria ulmifolia* | L | Zul/Cañ | 02866 RG |
| *Annonaceae* | *Xylopia aromatica* | YL | Amaz/Man | 14612 AF |
| *Annonaceae* | *Xylopia aromatica* | RB | Amaz/Man | 14612 AF |
| *Annonaceae* | *Xylopia aromatica* | OB | Amaz/Man | 14612 AF |
| *Annonaceae* | *Xylopia aromatica* | TW | Amaz/Man | 14612 AF |
| *Annonaceae* | *Xylopia aromatica* | L | Amaz/Man | 14612 AF |
| *Apocynaceae* | *Couma macrocarpa* | RB | Amaz/Man | 00550 BM |
| *Apocynaceae* | *Macoubea guianensis* | YL | Amaz/Man | 00582 BM |
| *Apocynaceae* | *Macoubea guianensis* | RB | Amaz/Man | 00582 BM |
| *Apocynaceae* | *Mandevilla scabra* | L/BR | Amaz/Man | 01442 BM |
| *Arecaceae* | *Euterpe precatoria* | YL | Amaz/Man | 01690 BM |
| *Arecaceae* | *Euterpe precatoria* | RH | Amaz/Man | 01690 BM |
| *Arecaceae* | *Euterpe precatoria* | FR | Amaz/Man | 01690 BM |
| *Arecaceae* | *Euterpe precatoria* | R | Amaz/Man | 01690 BM |
| *Arecaceae* | *Oenocarpus bataua* | ST | Amaz/Man | 01524 BM |
| *Arecaceae* | *Oenocarpus bataua* | R | Amaz/Man | 01524 BM |
| *Asclepiadaceae* | *Calotropis gigantea* | L | Coj/Gir | 02085 RG |
| *Asteraceae* | *Chromolaena odorata* | L | Coj/Gir | 23884 AF |
| *Asteraceae* | *Espeletia schultzii* | L | Mér/Lib | 24966 AF |
| *Asteraceae* | *Tithonia diversifolia* | L | Mir/Sal | 18543 AF |
| *Bignoniaceae* | *Jacaranda copaia* | ST | Amaz/Man | 22150 AF |
| *Bignoniaceae* | *Jacaranda copaia* | R | Amaz/Man | 22150 AF |
| *Bignoniaceae* | *Jacaranda copaia* | RB | Amaz/Man | 22150 AF |
| *Bignoniaceae* | *Jacaranda copaia* | YL | Amaz/Man | 22150 AF |
| *Bignoniaceae* | *Jacaranda copaia* | L | Amaz/Man | 22150 AF |
| *Bixaceae* | *Cochlospermum orinocense* | ST | Amaz/Man | 01473 BM |
| *Bixaceae* | *Cochlospermum orinocense* | FL | Amaz/Man | 01473 BM |
| *Bixaceae* | *Cochlospermum orinocense* | L | Amaz/Man | 01473 BM |
| *Bixaceae* | *Cochlospermum vitifolium* | BA | Coj/Gir | 02128 RG |
| *Bixaceae* | *Cochlospermum vitifolium* | L | Coj/Gir | 02128 RG |
| *Boraginaceae* | *Heliotropium Indicum* | L | Zul/Cañ | 02775 RG |
| *Bromeliaceae* | *Bromelia goeldiana* | FR | Amaz/Man | 01488 BM |
| *Bromeliaceae* | *Bromelia goeldiana* | L | Amaz/Man | 01488 BM |
| *Burseraceae* | *Protium crassipetalum* | BA | Amaz/Man | 01409 BM |
| *Burseraceae* | *Protium heptaphyllum* | L | Amaz/Man | 16278 AF |
| *Burseraceae* | *Protium heptaphyllum* | RB | Amaz/Man | 16278 AF |
| *Burseraceae* | *Protium heptaphyllum* | BR | Amaz/Man | 16278 AF |
| *Burseraceae* | *Protium heptaphyllum* | OB | Amaz/Man | 16278 AF |
| *Burseraceae* | *Protium unifoliolatum* | BA | Amaz/Man | 01313 BM |
| *Burseraceae* | *Protium unifoliolatum* | L | Amaz/Man | 01313 BM |
| *Caesalpiniaceae* | *Senna obtusifolia* | L/FL | Bol/Suc | 01714 BM |
| *Caesalpiniaceae* | *Senna silvestris* | FL | Amaz/Man | 00507 BM |
| *Caesalpiniaceae* | *Senna silvestris* | BA | Amaz/Man | 00507 BM |
| *Caesalpiniaceae* | *Senna silvestris* | FR | Amaz/Man | 00507 BM |
| *Caesalpiniaceae* | *Senna silvestris* | L | Amaz/Man | 00507 BM |
| *Clusiaceae* | *Vismia cayennensis* | L | Amaz/Man | 01484 BM |
| *Clusiaceae* | *Vismia cayennensis* | ST | Amaz/Man | 01484 BM |
| *Clusiaceae* | *Vismia guianensis* | L | Amaz/Man | 01523 BM |
| *Clusiaceae* | *Vismia guianesis* | BA | Amaz/Man | 01523 BM |
| *Clusiaceae* | *Vismia lindeniana* | L | Mon/Car | 02579 RG |
| *Costaceae* | *Costus scaber* | L | Amaz/Man | 01313 BM |
| *Dilleniaceae* | *Curatella americana* | YL | Amaz/Man | 01053 BM |
| *Dilleniaceae* | *Curatella americana* | FL | Amaz/Man | 01053 BM |
| *Dilleniaceae* | *Curatella americana* | L | Amaz/Man | 01053 BM |
| *Dilleniaceae* | *Curatella americana* | BA | Amaz/Man | 01053 BM |
| *Euphorbiaceae* | *Croton cuneatus* | L/FR | Amaz/Man | 01246 BM |
| *Euphorbiaceae* | *Croton cuneatus* | YL | Amaz/Man | 01246 BM |
| *Euphorbiaceae* | *Croton cuneatus* | RB | Amaz/Man | 01246 BM |
| *Euphorbiaceae* | *Croton cuneatus* | OB | Amaz/Man | 01246 BM |
| *Euphorbiaceae* | *Croton cuneatus* | RB | Amaz/Man | 01246 BM |
| *Flacourtiaceae* | *Lindackeria paludosa* | BA | Amaz/Man | 00937 BM |
| *Gnetaceae* | *Gnetum nodiflorum* | BR | Amaz/Man | 01316 BM |
| *Gnetaceae* | *Gnetum nodiflorum* | L | Amaz/Man | 01316 BM |
| *Gnetaceae* | *Gnetum nodiflorum* | ST | Amaz/Man | 01316 BM |
| *Humiriaceae* | *Humiria balsamifera* | L | Amaz/Man | 00189 BM |
| *Humiriaceae* | *Humiria balsamifera* | BA | Amaz/Man | 00189 BM |
| *Lamiaceae* | *Hyptis dilatata* | L/BR | Amaz/Man | 00507 BM |
| *Lamiaceae* | *Hyptis dilatata* | R | Amaz/Man | 00507 BM |
| *Lamiaceae* | *Hyptis dilatata* | FR | Amaz/Man | 00507 BM |
| *Lecythidaceae* | *Eschweilera parvifolia* | BR | Amaz/Man | 01441 BM |
| *Lecythidaceae* | *Eschweilera parvifolia* | RB | Amaz/Man | 01441 BM |
| *Lecythidaceae* | *Eschweilera parvifolia* | L | Amaz/Man | 01441 BM |
| *Malpighiaceae* | *Byrsonima crassifolia* | L | Amaz/Man | 00485 BM |
| *Malpighiaceae* | *Byrsonima crassifolia* | BA | Amaz/Man | 00485 BM |
| *Malpighiaceae* | *Byrsonima crassifolia* | FR | Amaz/Man | 00485 BM |
| *Malpighiaceae* | *Byrsonima crassifolia* | RB | Amaz/Man | 00485 BM |
| *Malpighiaceae* | *Byrsonima crassifolia* | FL | Amaz/Man | 00485 BM |
| *Meliaceae* | *Melia azedarach* | L/FL | Zul/Cañ | 02870 RG |
| *Mimosaceae* | *Parkia pendula* | BA | Amaz/Man | 00414 BM |
| *Moraceae* | *Ficus crocata* | L | Coj/Gir | 02636 RG |
| *Papaveraceae* | *Boconia integrifolia* | L/ST | Mér/CE | 25014 AF |
| *Piperaceae* | *Piper arboreum* | L | Amaz/Man | 16571 AF |
| *Piperaceae* | *Piper marginatum* | ST | Amaz/Man | 00485 BM |
| *Piperaceae* | *Piper marginatum* | L | Amaz/Man | 00485 BM |
| *Piperaceae* | *Piper san-vicentense* | L | Mér/AC | 25263 AF |
| *Polypodiaceae* | *Polypodium aureum* | RH | Amaz/Man | 00834 BM |
| *Polypodiaceae* | *Polypodium aureum* | L | Amaz/Man | 00834 BM |
| *Rubiaceae* | *Hamelia patens* | L | Amaz/Man | 01188 BM |
| *Rubiaceae* | *Psychotria poeppigiana* | L | Amaz/Man | 01046 BM |
| *Rubiaceae* | *Psychotria poeppigiana* | BR | Amaz/Man | 01046 BM |
| *Rubiaceae* | *Psychotria poeppigiana* | FL | Amaz/Man | 01046 BM |
| *Rubiaceae* | *Psychotria poeppigiana* | R | Amaz/Man | 01046 BM |
| *Rubiaceae* | *Uncaria guianensis* | BA | Amaz/Man | 01525 BM |
| *Rubiaceae* | *Warscewiczia coccinea* | L | Amaz/Man | 01316 BM |
| *Rubiaceae* | *Warscewiczia coccinea* | ST | Amaz/Man | 01316 BM |
| *Siparunaceae* | *Siparuna guianensis* | L | Amaz/Man | 14904 AF |
| *Siparunaceae* | *Siparuna guianensis* | R | Amaz/Man | 14904 AF |
| *Sterculiaceae* | *Guazuma ulmifolia* | L/FL | Zul/JMS | 02866 RG |
| *Sterculiaceae* | *Waltheria indica* | WP/-R | Bol/Suc | 16607 AF |
| *Theophrastaceae* | *Clavija lancifolia* | ST | Amaz/Man | 00474 BM |
| *Tiliaceae* | *Triunfetta semitriloba* | L/FR | Coj/Gir | 02104 RG |
| *Vochysiaceae* | *Vochysia ferruginea* | BR | Amaz/Man | 01286 BM |
| *Vochysiaceae* | *Vochysia ferruginea* | YL | Amaz/Man | 01286 BM |
| *Vochysiaceae* | *Vochysia ferruginea* | ST | Amaz/Man | 01286 BM |
| *Vochysiaceae* | *Vochysia ferruginea* | L | Amaz/Man | 01286 BM |
| *Vochysiaceae* | *Vochysia tilletii* | L | Amaz/Man | 01449 BM |
| *Vochysiaceae* | *Vochysia tillettii* | RB | Amaz/Man | 01449 BM |
| *Vochysiaceae* | *Vochysia venezuelana* | FL | Coj/Gir | 02638 RG |
| *Vochysiaceae* | *Vochysia venezuelana* | L | Coj/Gir | 02638 RG |

BA - bark, BR - branch, FL - flower, FR - fruit, L - leaf, L/BR - whole plant (leaf, branch without flower), L/FL - leaf and flower, L/FR - leaf and fruit, L/ST - leaf and stem, R - root, RB - internal bark (root), RH - stem (rhizome), ST - stem, TW - twig, WP - whole plant, WP/-FL - whole plant (without flower), WP/-R - whole plant without root, YL - young leaf, OB- outer bark.
